# Identify potential allelochemicals from *Humulus scandens* (Lour.) Merr. root extracts that induce allelopathy on *Alternanthera philoxeroides* (Mart.) Griseb.

**DOI:** 10.1038/s41598-021-86656-7

**Published:** 2021-03-29

**Authors:** Lichao Wang, Yao Liu, Xiaomin Zhu, Zhen Zhang, Xueqi Huang

**Affiliations:** School of Resources and Environment, Anhui Agricultural University, Hefei, 230036 China

**Keywords:** Community ecology, Invasive species

## Abstract

Although it is well-documented that invasion of invasive plants is promoted with allelopathic effects by inhibiting the growth and phenotypic performance of native plants, little is known conversely. In this study, the allelopathy effects of a native plant, *Humulus scandens* (Lour.) Merr., on a typical invasive species *Alternanthera philoxeroides* (Mart.) Griseb., was investigated by exposing *A. philoxeroides* seedlings to three chemical solvent extracts (i.e., petroleum ether extract (PE), ethyl acetate extract (EE), and n-butanol extract (NE) of *H. scandens* root (HR). The three chemical extracts inhibited the growth, stem length, node number, leaf number, leaf area, and root number, and increased malondialdehyde (MDA) content of *A. philoxeroides* seedlings, which indicated that the extracts inhibited the plant growth by damaging the membrane system of leaves. And the synthetical effect of allelopathy (SE) index indicated that EE had the greatest inhibition on the growth of *A. philoxeroides*. Fifty compounds were identified from the three extracts of HR using GC–MS analysis, among which 5 compounds (dibutyl phthalate, stigmasta-3,5-diene, 2,6-Di-tert-butylphenol campesterol, and neophytadiene) were identified from *H. scandens* root extracts for the first time. And n-hexadecanoic acid exists in all three extracts. The findings of the present study provide a novel method to potentially control the invasion of *A. philoxeroides*. However, field monitoring under natural conditions would be necessary to confirm in practice the results obtained with the bioassays.

## Introduction

Bioinvasion has become a serious environmental problem in the world in general and is considered as the second biggest threat to biodiversity. *Alternanthera philoxeroides* (Mart.) Griseb. (an *Amaranthaceae* family member, generally named alligator weed), is a worldwide invasive plant species^1^ of which invasion was reported in 32 different countries^2–4^. It grows well in terrestrial, aquatic, hygrophytic and other habitats^5^. *A. philoxeroides* was initially introduced into China as animal feed in the 1930s because of its fast growth, high photosynthetic rate^6–8^ and high nitrogen utilization rate. However, *A. philoxeroides* is currently considered as a significant threat to plant diversity^9,10^ because it is highly competitive to replace herbage and other plant species^11,12^, and is aggressive against cotton, corn, rice, soybean and a variety of vegetables^13,14^. Its asexual reproduction enables it to easily create new infection by stem fragmentation in most invaded areas^15,16^. Therefore, it has become a serious issue to control this invasive species.

Three principal means are generally utilized to control the alligator weed, being physical^17^, chemical^18,19^ and biological removal^20^. Physical methods are mainly to remove invasive plants by manual and mechanical methods; chemical methods are to spray chemical herbicides such as glyphosate to cause plant death; biological methods are mainly to control plant growth by natural enemies, soil animals and soil microorganisms. These methods are usually suffering from expensive costs^4^, lack of durability^19^, risks of accelerating invasion^21^ and producing herbicide-resistant weeds^22^, leading to poor efficiency of invasion control. However, allelopathy found its role in successful replacement control of invasive weeds^23,24^, consequently being introduced to combat the challenges of environmental pollution and herbicide resistance development^23,25^. Simultaneously, allelochemicals could be produced and degraded under natural conditions, avoiding the risks of secondary contamination during chemical control. A diverse array of allelochemicals are produced by plants, such as phenolic compounds, terpenoids, glycosteroids and alkaloids^26^. These chemicals are released by volatilization, leaching, root exudation and decomposition^27,28^. Rhizosphere biochemistry that is shaped by allelopathy may drive geographic co-evolutionary trajectories, affecting the coexistence of species and the development of plant communities, ultimately resulting in an invasion control^29^.

So far, it is generally accepted in research that invasive plants are able to inhibit the growth of native plants mainly by allelopathy effects^30^. For instance, fresh shoot aqueous extract of *Tithonia diversifolia*, an invasive species, significantly inhibited the radicle and plumule lengths of the maize (*Zea mays* L.) seedlings^31^. Nevertheless, the potential allelopathy effects of native species on alligator weed are rarely investigated in China. One of the limited study showed that extracts of *Phragmites australis* (Cav.) Trin inhibited the growth of invasive plant *A. philoxeroides*, providing a potential means to control this invasive plant^24^. Using allelopathy of native plants to control invasive plants may become a potential novel method of invasion control. *Humulus scandens* (Lour.) Merr. (belonging to the Moraceae family) is widely distributed in China, mainly growing at the edge of ditch, wasteland, ruins and forests^32^. *H. scandens* is more competitive than *A. philoxeroides* in the field and laboratory^33^.

The present study focused on two questions: (1) Does *H. scandens* root extract have allelopathic inhibition on the growth of *A. philoxeroides*? (2) What are the main secondary metabolites in *H. scandens* that potentially have allelopathy effects on *A. philoxeroides*? By answering these important questions, this study aimed to develop a potential method to control the invasion of *A. philoxeroides* by making use of the allelopathy effects of native plants if they exist.

## Results

### Influence of *Humulus scandens* root (HR) extracts on morphology index of *A. philoxeroides* seedlings

**Figure 1:**
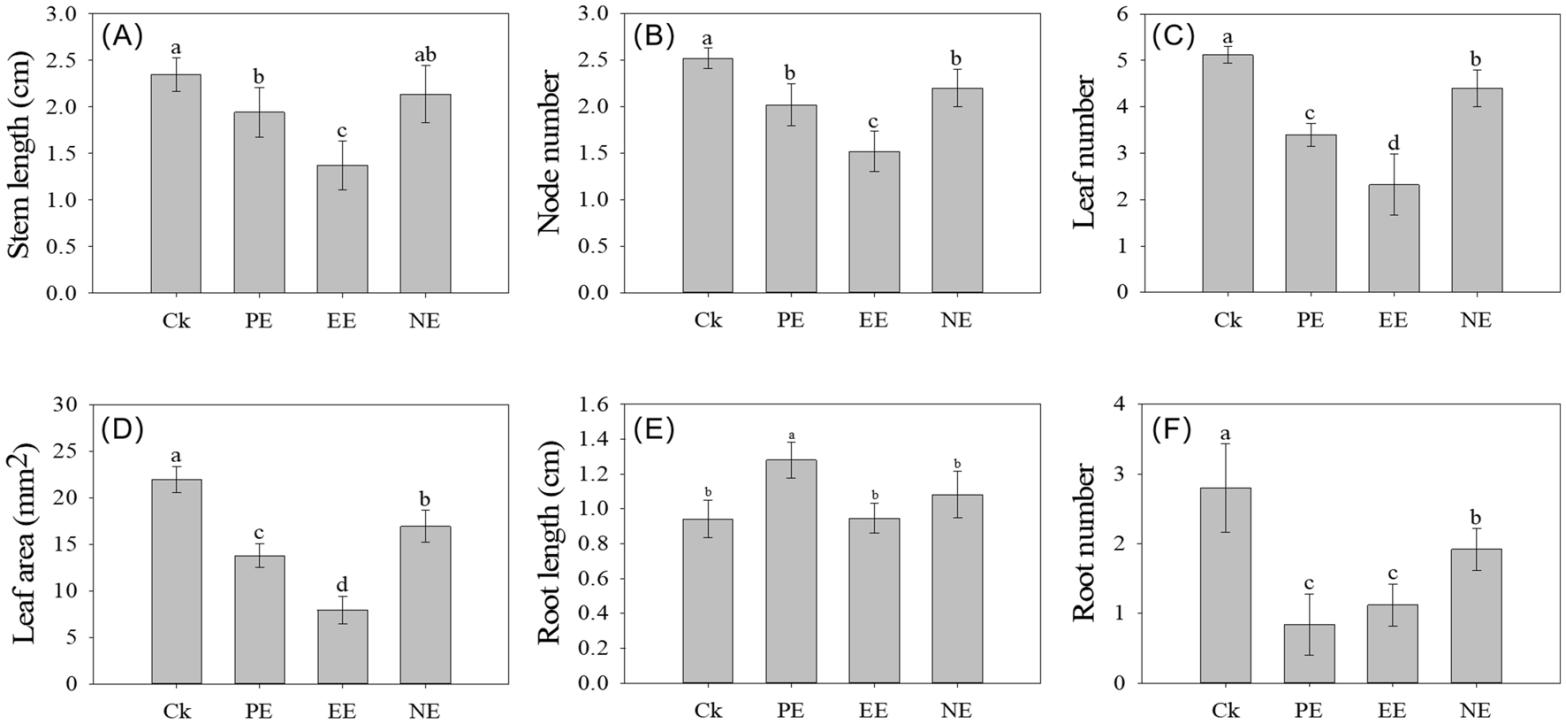
The effects of different subsurface extracts on the growth of *A. philoxeoides*. (**A**) stem length and (**B**) node number of ramets and (**C**) leaf number of ramets and (**D**) leaf area and (**E**) root length and (**F**) root number of *A. phlioxeoldes* (from left to right, the columns are control, Petroleum ether (PE), ethyl acetate (EE) and n-Butanol (NE)). The bars in the figure stand for the Std. Deviation of the replications (n = 5). The treatments with the same letter are not significantly difference at 0.05 level.

Different chemical extracts of HR posed a significant influence on the growth of *A. philoxeroides* (Fig. 1). Stem length, node number and leaf number decreased initially and then increased along with an increase in the extractant polarity. There were significant differences in the morphology index of *A. philoxeroides* between treatments and control, namely stem length (*F* = 13.16, *P* < 0.001), node number (*F* = 23.34, *P* < 0.001), leaf number (*F* = 43.396, *P* < 0.001), leaf area (*F* = 144.7, *P* < 0.001) and root number (*F* = 20.128, *P* < 0.001). The solvent extractions of petroleum ether extract (PE) and ethyl acetate extract (EE) had significant inhibitory effects on stem length (*P* = 0.026, *P* < 0.001), node number (*P* = 0.001, *P* < 0.001), leaf number (*P* < 0.001, *P* < 0.001) and leaf area (*P* < 0.001, *P* < 0.001), which reduced 17–83% than those in control. However, the PE extraction promoted the root length of *A. Philoxeroides* (*P* < 0.001), which increased 36% than that in control (Fig. 1E). The n-butanol extract (NE) extraction significantly inhibited the node number (*P* = 0.019) by 13%, while it has no significant effect on stem length (*P* = 0.22) compared with control.

### Effects of HR extracts on biomass of *A. philoxeroides* seedlings

Different chemical extracts of HR had significant inhibitory effects on the total biomass (*F* = 24.315, *P* < 0.001) and aboveground biomass (*F* = 15.831, *P* < 0.001) of *A. Philoxeroides* as well, reducing the biomass by 16–68% compared with control (Table 1). The extracts EE and NE significantly reduced the belowground biomass (*P* < 0.001, *P* = 0.029) by 72% and 29%, respectively, while PE extract significantly increased it (*P* < 0.001) by 37%. The PE and EE extracts had significant inhibitory effect on the leaf area ratio (LAR), reducing it by 53% and 50% respectively. The PE extract significantly enhanced the root/shoot ratio of *A. philoxeroides* (*P* = 0.004).

**Table 1:** Effects of three chemical extracts on the biomass, LAR and Root/shoot ratio of *A.philoxeroides* (Mean ± SD)*.*

	Aboveground biomass	Belowground biomass	Total biomass	Leaf area ratio(LAR)	Root/shoot ratio
Control	21.25 ± 5.73a	3.65 ± 0.66b	24.9 ± 5.51a	4.67 ± 0.96a	0.18 ± 0.06b
Petroleum ether (PE)	15.8 ± 2.34b	5 ± 0.42a	20.8 ± 2.01ab	2.26 ± 0.19b	0.33 ± 0.07a
Ethyl acetate (EE)	7.04 ± 0.93c	1.02 ± 0.29d	8.06 ± 1.01d	2.37 ± 1.03b	0.15 ± 0.05b
N-Butanol (NE)	14.83 ± 2.03b	2.58 ± 0.69c	17.46 ± 2.63c	4.34 ± 0.86a	0.17 ± 0.03b

The treatments with the same letter are not significantly different at the 0.05 level.

### Impacts of HR extracts on peroxidases activity of* A. philoxeroides* leaves

**Figure 2:**
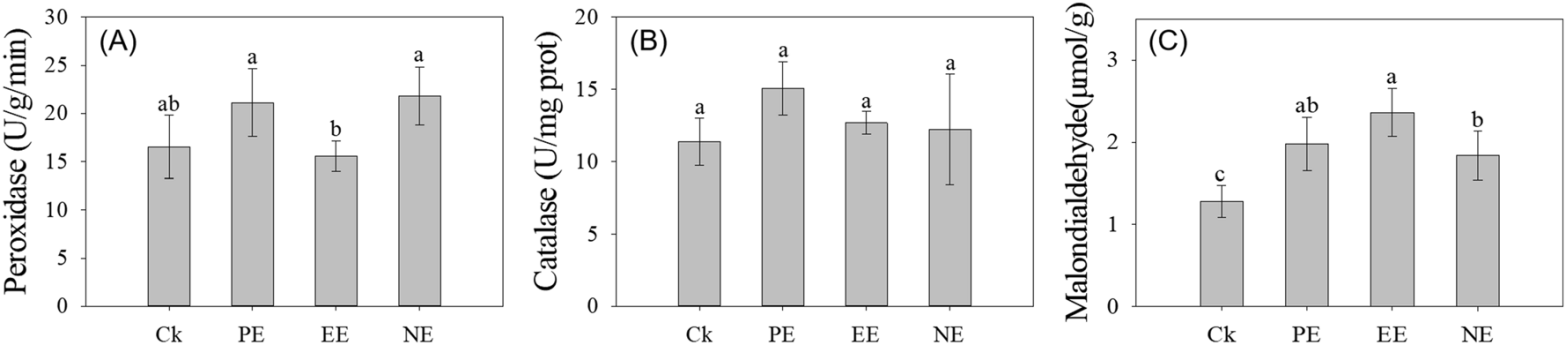
The effect of the three chemical extracts on the leaf enzymes*,* (**A**) POD activity and (**B**) CAT activity and (**C**) MDA content, of *A. philoxeroides* (from left to right, the bars are control, PE, EE and NE, respectively). The treatments with the same letter are not significantly difference at 0.05 level.

The three chemical extracts had significant effects on the leaf POD activity (*F* = 5.81, *P* = 0.007) and MDA content (*F* = 12.75, *P* < 0.001) of *A. philoxeroides* (Fig. 2A,C), while they did not induce any significant differences in CAT activity (*F* = 2.35, *P* = 0.111) (Fig. 2B). The PE and NE extracts significantly stimulated the POD activity (*P* = 0.025 and *P* = 0.012) by 28% and 32%, respectively. The MDA content of *A. philoxeroides* leaf with PE (*P* = 0.001), EE (*P* < 0.001) and NE (*P* = 0.006) extracts treatment significantly increased by 44–85% compared with control.

### Identification of potential allelochemicals

A total of 50 compounds were identified from the three extracts (Table 2), mainly being phenols, terpenes, alkaloids. Among them, 30 compounds were identified from petroleum ether extracts, which were 16 phenols and their derivatives, 7 terpenes, 7 alkaloids. The highest relative content was dibutyl phthalate (14.77%), followed by n-Hexadecanoic acid (13.99%), 9,12,15-Octadecatrienoic acid, (Z,Z,Z)- (12.33%), 9,12-Octadecadienoic acid (Z,Z)- (11.79%). Twenty-six compounds were identified from ethyl acetate extract, including 13 phenols and their derivatives, 5 terpenes, 3 alkaloids. Among these 26 chemicals, the highest relative content was presented by methyl palmitate (11.8%), followed by stigmasta-3,5-diene (8.75%), (Z,Z)-Octadeca-9, 12-dienoic acid (7.62%), n-Hexadecanoic acid (7.38%), 9,12-Octadecadienoic acid (Z,Z)-methyl ester (6.13%). Twelve compounds were identified from n-butanol extract, being 5 phenols and their derivatives, 4 terpenes, and 2 alkaloids. Among these 12 chemicals stigmastane-3,6-dione,(5à) (6.73%), n-Hexadecanoic acid (6.29%), and á-sitosterol (5.63%) were with the highest relative content.

**Table 2:** Compounds in petroleum ether extracts that were identified by GC/MS.

	Compound Name	Petroleum ether extract (PE) Content %	Ethyl acetate extract (EE) Content %	N-Butanol extract (NE) Content %
1	n-Hexadecanoic acid	13.99	7.38	6.29
2	Tetradecanoic acid	0.81	0.55	–
3	Neophytadiene	0.52	0.89	–
4	Hexadecanoic acid, methyl ester	2.95	11.8	–
5	9,12-Octadecadienoic acid (Z,Z)-, methyl ester	1.66	6.13	–
6	9,12,15-Octadecatrienoic acid, methyl ester, (Z,Z,Z)-	1.21	4.99	–
7	9,12-Octadecadienoic acid (Z,Z)-	11.79	7.62	–
8	9,12,15-Octadecatrienoic acid, (Z,Z,Z)-	12.33	4.37	–
9	(E)-13-Docosenoic acid	0.41	0.62	–
10	Eicosanoic acid	1.38	1.48	–
11	Stigmasterol	0.66	2.27	–
12	Stigmasta-5,22-dien-3-ol, acetate, (3á)-	2.7	1.78	–
13	Stigmasta-3,5-diene	2.29	8.75	–
14	1-Heptatriacotanol	0.4	–	2.06
15	Stigmastane-3,6-dione, (5à)-	0.73	–	6.73
16	á-Sitosterol	0.46	–	5.63
17	Betulinaldehyde	–	0.68	1.7
18	1,3-Dioxolane, 4,5-dimethyl-2-pentadecyl-	–	–	0.73
19	Ethanol, 2-[4-(1,1-dimethylethyl)phenoxy]-	–	0.48	–
20	Benzoic acid, 2-hydroxy-, butyl ester	–	–	0.55
21	1,2,3,4-Tetrahydroisoquinolin-6-ol-1-carboxylic acid	0.55	–	–
22	2,6-Di-tert-butylphenol	–	0.69	–
23	2-Pentadecanone, 6,10,14-trimethyl-	0.56	–	–
24	trans-13-Octadecenoic acid	0.55	–	–
25	17-Octadecynoic acid	–	0.72	–
26	Pentadecanoic acid	0.96	–	–
27	1,2-Benzenedicarboxylic acid, bis(2-methylpropyl) ester	1.56	–	–
28	Ethanol, 2-(9-octadecenyloxy)-, (Z)-	–	0.87	–
29	9-Hexadecenoic acid, methyl ester, (Z)-	–	0.43	–
30	Pentadecanoic acid, 13-methyl-, methyl ester	–		1.22
31	1,2-Benzenedicarboxylic acid, butyl octyl ester	–	0.52	–
32	Methyl stearate	–	1.26	–
33	Octadecanoic acid	–	2.02	–
34	Octadecanoic acid	2.67	–	–
35	Octadecanoic acid, ethyl ester	0.69	–	–
36	Bis(2-ethylhexyl) phthalate	2.24	–	–
37	Glycidol stearate	0.7		
38	Hexadecanoic acid, ethyl ester	0.83		
39	Hexanedioic acid, bis(2-ethylhexyl) ester	1.58		
40	Cholest-5-en-3-one	0.92		
41	Lup-20(29)-en-3-one	2.59		
42	4,22-Stigmastadiene-3-one	0.64		
43	Eicosanoic acid, methyl ester		0.87	
44	Docosanoic acid, methyl ester		1.18	
45	Trilinolein		0.8	
46	Campesterol		1.43	
47	Betulin			0.52
48	Ursodeoxycholic acid			1.34
49	Hexadecanoic acid, butyl ester			1.5
50	Oleic acid, eicosyl ester			2.21

In this study, 5 compounds were identified from *H. scandens* root extracts for the first time, which were dibutyl phthalate, stigmasta-3,5-diene, 2,6-Di-tert-butylphenol campesterol, and neophytadiene. And n-hexadecanoic acid exists in all three extracts.

### Evaluation of allelopathic effects

The three chemical extracts inhibited the stem length, node number, leaf number, leaf area, above- and total biomass of *A. philoxeroides* (*RI* < 0, Table 3). The allelopathic inhibition of EE extract on stem, leaf and biomass of *A. philoxeroides* was significantly greater than that of PE and NE extracts, while EE extract promoted root length (*RI* > 0). The synthetical effect of allelopathy (SE) index indicated that EE had the greatest inhibition on the growth of *A. philoxeroides* (*RI* = -0.539), followed by PE (*RI* = -0.209) and NE (*RI* = -0.197).

**Table 3:** The allelopathic effects of the extracts of *H.scandens* on *A.philoxeroides.*

	Stem length	Node number	Root length	Root number	Leaf number	Leaf area	Aboveground biomass	Belowground biomass	Total biomass	SE
Petroleum ether	− 0.17	− 0.20	0.26	− 0.70	− 0.34	− 0.58	− 0.26	0.27	− 0.16	− 0.209
Ethyl acetate	− 0.41	− 0.40	0.00	− 0.60	− 0.55	− 0.83	− 0.67	− 0.72	− 0.68	− 0.539
N-Butanol	− 0.09	− 0.13	0.13	− 0.31	− 0.14	− 0.34	− 0.30	− 0.29	− 0.30	− 0.197

## Discussion

Allelopathy is ubiquitously existing among plant species, generally being tested with the effects on the plant seedling growth^34^. Common native species, for instance *pueraria lobata* and *paederia scandens*, depressed growth of *Ipomoea cairica*^35^. Allelochemicals inhibited protein synthesis^36^ and cell division and elongation^37^, consequently affecting plant growth and development. However, the study of allelopathy mainly focuses on the allelopathy of invasive plants to native plants, while the opposite study may become a new way to control invasive weeds. The present study identified an inhibitory allelopathy effect of a native plant species *H. scandens* on *A. philoxeroides*, being revealed by the fact that three chemical extracts of HR inhibited the seedling growth of *A. philoxeroides* (*SE* < 0, Table 3). Inhibition of shoot growth of *A. philoxeroides* was previously identified as well with extracts, residues and allelochemicals from different plants and fungi^4^. Biomass is one of the main important factors controlling the spread of *A. philoxeroides*^21,38^. The regrowth capacity of alligator weed was weakened by removing and destroying its above- and below-ground biomass^4^. An inhibited aboveground biomass, belowground biomass, total biomass and the leaf area of *A. philoxeroides* were found in our study with exposure to ethyl acetate extract of HR (*RI* = − 0.67, − 0.72, − 0.68, − 0.83, respectively). Allocation indicates the investment of plants in resource utilization. Therefore, the reduced biomass of *A. philoxeroides* would likely reduce the ability of this invasive plant to absorb nutrients and capture light energy.

Under optimal conditions, the balance between reactive oxygen species (ROS) formation and consumption is tightly controlled by antioxidant enzymes and redox metabolites^39,40^. However, allelochemicals were able to induce cell membrane permeability (for example, of saccharomycetes, sugar beet, maize and so on)^28^ and oxidative stress^41^. In the present study, an increased POD activity in the leaves of *A. philoxeroides* with the treatment of PE and NE extracts (Fig. 2A) indicated an accelerated H_2_O_2_ stress that was potentially induced by the extracts of HR. Phenolic compounds caused oxidative damage in peanut seedlings and increased the contents of catalase (CAT) and peroxidase (POD) in leaves compared with the control, which is mutually confirmed by this study^42^. MDA is one of the lipid peroxidation products of biofilm system^43,44^. The higher the content of MDA in the plant, the more obvious the degree of injury^45^. An increased MDA content could damage the membrane system of leaves and consequently inhibit the growth of seedlings^46^. In this study, compared with Ck, MDA content in leaves of *A. philoxeroides* treated with three extracts (PE, EE and NE) increased significantly (Fig. 2C), and EE treatment reached the highest. In conclusion, *H. scandens* may release allelochemicals, which may have negative effects on the ROS of *A. philoxeroides* leaves, thus inhibiting its normal growth.

There are many studies on the chemical constituents of *H. scandens*, more in the field of traditional Chinese medicine. Compounds β-sitosterol, carotene, apigenin, daucesterol, stigmast-3,6-dione, n-hexadecanoic, linoleic acid and stigmasterol were isolated and identified from the whole plant of *H. scandens*^42,47^. Compounds were obtained from the ethyl acetate fraction of methanol extract stems of *H. scandens* and identified as cis-N-p-coumaroyltyramine, N-cis-feruloyltyramine, trans-N-p-coumaroyltyramine, Vomifoliol^48^. In this study, 5 compounds were isolated from *H. scandens* root extracts for the first time, which were dibutyl phthalate, stigmasta-3,5-diene, 2,6-Di-tert-butylphenol campesterol, and neophytadiene. And n-hexadecanoic acid exists in all three extracts. The compound “stigmasta-3,5-diene” is used in medical research and has biological activity against certain inflammatory diseases^49^. Some scholars utilized GC–MS to separate from Solidago Canadensis the compounds 2,6-Di-tert-butylphenol^50^, which showed certain allelopathy to the growth of the *Solanum melongena* L. seedings^51,52^. Compound campesterol shows various degrees of allelopathic activity on common weeds, such as red chilli and legumes^53–55^. Compound dibutyl phthalate has certain allelotoxicity to tobacco seedlings and the growth of Microcystis aeruginosa^56,57^.

The results showed that *H. scandens* root extracts significantly inhibited the growth of alligator weeds, mainly being indicated by physiological, biochemical and morphological indices. At the same time, 50 compounds were identified by GC–MS, among which 5 compounds were identified for the first time from *H. scandens* root extracts. However, there are still some limitation in this study. For instance, in the laboratory, allergies are not disturbed; but in the natural environment, it is affected by climate, temperature, soil animals, soil microorganisms and other factors. Therefore, the potential of allelopathy in the prevention and control of alligator weed should be elaborated in future research based on the actual environment.

## Materials and methods

### Experimental materials

Plants of *A. philoxeroides* and *H. scandens* were collected from the campus of Anhui Agriculture University in China (N31°52′, E117°16′). Fresh *H. scandens* roots were dried to constant weight under shade, and ground to fine powder passing through 40 mesh sieve, then put in a desiccator. The *A. philoxeroides* plants were cut into several cuttings, each with one node. The regents 95% ethanol, petroleum ether, ethyl acetate, and n-butanol were analytical pure, purchased from Jinan Century Tongda Chemical Co., Ltd.

### Preparation and isolation of the HR extracts

HR extracts were prepared according to Alara and Abdurahman extraction methods^58,59^ with slight modification. Ten gram (total two kilogram) of HR was tightly wrapped with one layer of filter paper and soxhlet extraction with 95% ethanol for 3 h. The extraction was then concentrated into paste using a rotary evaporator (Rotavapor RE-52A coupled with SHE-III circulating water vacuum pump, Shanghai), then it was dissolved in distilled water and extracted three times with petroleum ether, n-butanol and ethyl acetate. The same components were mixed and concentrated, resulting in three concentrated extraction, being petroleum ether extract (PE), ethyl acetate extract (EE) and n-butanol extract (NE), respectively. The concentrated extractions were then stored in seal at 4 ℃ in dark place.

### Exposure of *A. philoxeroides* to three organic extracts

*A. philoxeroides* and the fractions of the concentrated extracts (PE, EE and NE, with a concentration of 2 mg mL^−1^) dissolved in distilled water were add into petri dishes (9 cm diameter) covered with two layers of filter paper. Dishes without any HR extracts were taken as the control (distilled water). Five plant tissues of *A. philoxeroides* were randomly placed in each dish. Each plant tissue contained one node that was 3 cm long and 0.4 cm in stem thick. After 15 days incubation in an incubator (temperature 28℃, light intensity 400 μmol·cm^−2^·s^−2^, 16/8 h light/dark), the plant tissues were collected for experimental analysis.

To verify the effects of HR extracts on the seeding development of *A. philoxeroides*, the morphology indices were determined as follows. Stem length and root length were measured directly with a ruler. The number of nodes, leaves and roots were counted. Plant biomass (aboveground and belowground) was measured following dried in oven at 65℃ for 48 h till constant weight^24^. Root/shoot ratio, leaf area ratio and leaf area were calculated as below^24,60^:

$$\begin{aligned} {\text{Root}}/{\text{shoot}}\;{\text{ratio}} & = {\text{Underground}}/{\text{Aboveground}}\;{\text{biomass}}; \\ {\text{Leaf}}\;{\text{area}} & = \pi *\left( {\text{Leaf length}} \right)*\left( {\text{Leaf width}} \right)/{4}; \\ {\text{Leaf}}\;{\text{area}}\;{\text{ratio}}\left( {{\text{LAR}}} \right) & = {\text{Leaf area}}/{\text{Total biomass}}; \\ \end{aligned}$$

Fresh leaves were separately collected to measure the related enzymes peroxidase (POD), catalase (CAT) and malondialdehyde (MDA) content with kits (Lai Er Bio-Tech). Use POD kit, CAT kit to measure leaf enzyme activity and MDA kit to measure leaf MDA content. Collect fresh clonal plant leaves, rinse 3 times with pure water, wipe dry, weigh 0.2 g and cut into a 2 mL centrifuge tube, add 1.8 mL phosphate buffer, crush with a high-throughput tissue grinder, and then use a centrifuge for 3000 rpm/min Centrifuge, take the supernatant and store at 4 °C for testing (the whole process of leaf treatment is performed under low temperature conditions).

### Evaluation of allelopathic effects

The allelopathic effect of the extract was determined as following^61^:

$$RI = \left\{ \begin{gathered} 1 - {C \mathord{\left/ {\vphantom {C {T\left( {T \ge C} \right)}}} \right. \kern-\nulldelimiterspace} {T\left( {T \ge C} \right)}} \hfill \\ {T \mathord{\left/ {\vphantom {T C}} \right. \kern-\nulldelimiterspace} C} - 1\left( {T < C} \right) \hfill \\ \end{gathered} \right.$$

where T represents growth response of test species treated with extracts and C represents growth response of the test species treated with distilled water (control). A positive RI value indicates that the extract promotes the seedling growth, whereas a negative RI value indicates that the extract inhibits the seedling growth.

Synthetical effect of allelopathy index (SE) was applied to evaluate the allelopathic effect by the average of several RIs and determined as following ^38^:

$${\text{SE}} = \left( \begin{gathered} RI_{{{\text{stem}}length}} + RI_{nodenumber} + RI_{{{\text{leafnumber}}}} + RI_{leafarea} + RI_{rootnumber} \hfill \\ + RI_{rootlength} + RI_{abovegroundbiomass} + RI_{belowgroundbiomass} + RI_{totalbiomass} \hfill \\ \end{gathered} \right)/9$$

### Identification of potential allelochemicals from the EE, PE and NE extractions

The extracted samples were dissolved in n-hexane (chromatographically pure) and analyzed by GC–MS (TRACE ISQ, Thermo Scientific). The injector temperature was 280℃. The initial column temperature was constant at 60℃ for 5 min, increased to 100 ℃ at a rate of 3.5 ℃/min for 5 min, then ramped to 200 ℃ at 8 ℃/min for 5 min. The temperature was then brought to 280℃ at a rate of 15 ℃/min and held until the end of the 15-min run. Helium was the carrier gas and the program was not divided.

Agilent data analysis software and NIST11 library were used to determine the retention time of chromatography, and peak area was used to calculate the content of the substance^62^. According to the 80% principle, compounds with library matching coefficient greater than or equal to 80% are used for analysis^63^.

### Data analysis

The experiment followed a completely randomized design, composed of three extracts (petroleum ether, ethyl acetate, and n-butanol) of *A. philoxeroides.* Data were tested for normality and homogeneity of variance. *ANOVA* was conducted on morphology and physiological indices with exudate treatments for *A. philoxeroides*. Differences between means were assessed with LSD’s and Duncan’s test (*P* < 0.05), using the SPSS v.21.0 for Windows. Graphs are performed by Sigmaplot 12.0.

## Supplementary Information

Supplementary Information.
