# Supplementary material for: Identify potential allelochemicals from Humulus scandens (Lour.) Merr. root extracts that induce allelopathy on Alternanthera philoxeroides (Mart.) Griseb
Source: Sci Rep. 2021 Mar 29;11:7068. doi: 10.1038/s41598-021-86656-7 (PMC8007610; doi:10.1038/s41598-021-86656-7)
Supplement: Supplementary file 1 — Supplementary Information. [file 41598_2021_86656_MOESM1_ESM.doc]

Identify potential allelochemicals from *Humulus scandens* (Lour.) Merr. root extracts that induce allelopathy on *Alternanthera philoxeroides* (Mart.) Griseb.

Lichao Wang, Yao Liu, Xiaomin Zhu, Zhen Zhang*, Xueqi Huang

School of Resources and Environment, Anhui Agricultural University, Hefei 230036, China.

***Authors for correspondence**:

Zhen Zhang (Z. Zhang);

Email address: [xjzhangzhen@163.com](mailto:xjzhangzhen@163.com)

**Supplementary material**

Fig.S1 Total ion current chromatogram of the different extracts (Petroleum ether extract (A), ethyl acetate extract (B) and n-Butanol extract (C)).
